# Comparison of immunohistochemistry with immunoassay (ELISA) for the detection of components of the plasminogen activation system in human tumour tissue

**DOI:** 10.1038/sj.bjc.6690245

**Published:** 1999-03

**Authors:** C M Ferrier, H H de Witte, H Straatman, D H van Tienoven, W L van Geloof, F J R Rietveld, C G J Sweep, D J Ruiter, G N P van Muijen

**Affiliations:** Department of Pathology, University Hospital Nijmegen, PO Box 9101, Nijmegen, 6500 HB, The Netherlands; Department of Chemical Endocrinology, University Hospital Nijmegen, PO Box 9101, Nijmegen, 6500 HB, The Netherlands; Department of Epidemiology, University Hospital Nijmegen, PO Box 9101, Nijmegen, 6500 HB, The Netherlands

**Keywords:** ELISA, immunohistochemistry, plasminogen activator system, correlation

## Abstract

Enzyme-linked immunosorbent assay (ELISA) methods and immunohistochemistry (IHC) are techniques that provide information on protein expression in tissue samples. Both methods have been used to investigate the impact of the plasminogen activation (PA) system in cancer. In the present paper we first compared the expression levels of uPA, tPA, PAI-1 and uPAR in a compound group consisting of 33 cancer lesions of various origin (breast, lung, colon, cervix and melanoma) as quantitated by ELISA and semi-quantitated by IHC. Secondly, the same kind of comparison was performed on a group of 23 melanoma lesions and a group of 28 breast carcinoma lesions. The two techniques were applied to adjacent parts of the same frozen tissue sample, enabling the comparison of results obtained on material of almost identical composition. Spearman correlation coefficients between IHC results and ELISA results for uPA, tPA, PAI-1 and uPAR varied between 0.41 and 0.78, and were higher for the compound group and the breast cancer group than for the melanoma group. Although a higher IHC score category was always associated with an increased median ELISA value, there was an overlap of ELISA values from different scoring classes. Hence, for the individual tumour cases the relation between ELISA and IHC is ambiguous. This indicates that the two techniques are not directly interchangeable and that their value for clinical purposes may be different. © 1999 Cancer Research Campaign


*British Journal of Cancer* (1999) **79**(9/10), 1534–1541
© 1999 Cancer Research Campaign
Article no. bjoc.1998.0245

### Comparison of immunohistochemistry with immunoassay (ELISA) for the detection of components of the plasminogen activation system in human tumour tissue

**CM Ferrier[1], HH de Witte[2], H Straatman[3], DH van Tienoven[2], WL van Geloof[1], FJR Rietveld[1], CGJ Sweep[2], DJ Ruiter[1] and GNP van Muijen[1]**

Departments of [1]Pathology, [2]Chemical Endocrinology and [3]Epidemiology, University Hospital Nijmegen, PO Box 9101, 6500 HB Nijmegen, The Netherlands

**Summary** Enzyme-linked immunosorbent assay (ELISA) methods and immunohistochemistry (IHC) are techniques that provide information on protein expression in tissue samples. Both methods have been used to investigate the impact of the plasminogen activation (PA) system in cancer. In the present paper we first compared the expression levels of uPA, tPA, PAI-1 and uPAR in a compound group consisting of 33 cancer lesions of various origin (breast, lung, colon, cervix and melanoma) as quantitated by ELISA and semi-quantitated by IHC. Secondly, the same kind of comparison was performed on a group of 23 melanoma lesions and a group of 28 breast carcinoma lesions. The two techniques were applied to adjacent parts of the same frozen tissue sample, enabling the comparison of results obtained on material of almost identical composition. Spearman correlation coefficients between IHC results and ELISA results for uPA, tPA, PAI-1 and uPAR varied between 0.41 and 0.78, and were higher for the compound group and the breast cancer group than for the melanoma group. Although a higher IHC score category was always associated with an increased median ELISA value, there was an overlap of ELISA values from different scoring classes. Hence, for the individual tumour cases the relation between ELISA and IHC is ambiguous. This indicates that the two techniques are not directly interchangeable and that their value for clinical purposes may be different.

**Keywords:** ELISA; immunohistochemistry; plasminogen activator system; correlation

A delicate interplay between the components of the plasminogen activation (PA) system influences the extent of proteolytic degradation of extracellular matrix. Five major components of the PA system are: urokinase-type and tissue-type plasminogen activator (uPA and tPA), plasminogen activator inhibitor type 1 and type 2 (PAI-1 and PAI-2) and a receptor for uPA (uPAR). In several tumour types increased levels of these components have been found, and their production is supposed to influence the invasive and metastasizing behaviour of tumour cells (de Vries et al, 1996; Andreasen et al, 1997). Clinical studies, mostly using enzyme-linked immunosorbent assay (ELISA) methods, emphasized the value of uPA as a prognostic marker and, in addition, showed the prognostic impact of other PA components in several cancer types (Kuhn et al, 1994; Duggan et al, 1995; Foekens et al, 1995; Ganesh et al, 1996; Schmitt et al, 1997). Another, although less validated, approach to investigate the prognostic significance of these components is immunohistochemistry (IHC) (Kobayashi et al, 1994; Mulcahy et al, 1994; Hsu et al, 1995; Heiss et al, 1996; Pappot et al, 1997; Umeda et al, 1997; Ruiter et al, 1998).

*Received 20 January 1998*
*Revised 29 July 1998*
*Accepted 10 August 1998*

*Correspondence to:* CM Ferrier

ELISA and IHC both have their specific advantages. Regarding ELISA for the PA components, the measurements are of established clinical value, standardization is being attained and a number of multicenter studies are already subject to quality assurance (Sweep et al, 1998). Furthermore, ELISA methods give an objective quantification of analyte levels, whereas IHC yields at best semi-quantitative information. However, IHC allows insight into tissue heterogeneity. Moreover, the distribution of an antigen over the different cell types, and therefore the clinical relevance of expression of an antigen by a particular cell type, can be studied. Another advantage of IHC is that it can often be performed on both frozen sections and routinely processed paraffin-embedded tissue (Ferrier et al, 1998).

In the present paper we compared semi-quantitatively scored IHC determinations with quantitative ELISA measurements for uPA, tPA, PAI-1 and uPAR in a panel of cancer lesions of different origin (breast, lung, colon, cervix and melanoma). In a second stage, more samples of breast carcinoma and melanoma were added, and the same kind of analysis was performed on these cancer types separately. ELISAs were performed on extracts of multiple thick tumour tissue sections and, to obtain the highest possible uniformity of test material, IHC was done on adjacent frozen sections. The degree of concordance of results found with the two techniques may illuminate the position of the two techniques regarding each other.

### MATERIALS AND METHODS

### Tissues

Tumour specimens that had been snap-frozen in liquid nitrogen immediately after surgery and stored at –80°C were obtained from the tissue bank of the Department of Pathology, University Hospital Nijmegen, The Netherlands. All specimens were derived from invasive primary or metastatic tumours, and contained at least 75% tumour tissue. In the first phase, samples from six lung carcinomas, six colon carcinomas, six carcinomas of the uterine cervix, six melanomas and nine breast carcinomas were investigated. Of these specimens, two sets of ten 20-µm thick sections were cut for analysis by ELISA. Adjacently, 4-µm thick sections were cut for standard haematoxylin–eosin staining and IHC. In the second phase, 17 more melanoma and 19 more breast carcinoma tissues were analysed. Of these samples only one set of ten 20-µm thick sections were cut for analysis by ELISA plus adjacent 4-µm thick sections for (immuno)histology.

### Tissue extraction

Pooled tissue fragments (10 × 20 µm) from one specimen were pulverized in the presence of 300 µl extraction buffer by means of a microdismembrator (B Braun Biotech International GmbH, Melsungen, Germany) at –170°C. After thawing on ice, the homogenates were centrifuged at 105 000 ***g*** for 1 h at 4°C and the supernatants were stored at –80°C until further analysis in the ELISAs.

For the set of 33 samples of various cancer types, two buffers were compared for their extraction efficiency: (i) a phosphate buffer: 10 mM $K_2HPO_4/KH_2PO_4$, 1.5 mM $K_2$-EDTA, 10 mM monothioglycerol, 10% (v $v^{-1}$) glycerol, 10 mM sodium molybdate (pH = 7.5) (Thorpe, 1987) and (ii) a detergent-containing Tris buffer: 0.1 M Tris-HCl (pH = 8.1), 1% (w $v^{-1}$) Triton X-114, 10 mM EDTA, 10 µg $ml^{-1}$ aprotinin (Behrendt et al, 1990).

For the extra melanoma and breast carcinoma samples, only the detergent-containing Tris buffer was used for extraction.

### ELISAs

Antigen levels of uPA, tPA and PAI-1 were determined using recently developed ELISAs (Grebenschikov et al, 1997). uPAR levels were determined using the ELISA as described by Rønne et al (1995). The ELISAs detect both the free forms and relevant complexes of the specific component with other components. Standards, controls and samples were all diluted in phosphate-buffered saline (PBS) containing 1% bovine serum albumin (BSA) (Fraction V; Boehringer Mannheim GmbH, Mannheim, Germany) and 0.1% (v $v^{-1}$) Tween-20. Incubation was performed overnight at 4°C. As a substrate 0.02% orthophenylenediamine (DAKO, Glostrup, Denmark)/0.01% hydrogen peroxide was used, and absorbances were read at 492 nm (reference wavelength 620 nm) with an automated ELISA reader (AR 2001, Anthos Labtec Instruments GmbH, Salzburg, Austria). All samples were assayed in duplicate. ELISA results were expressed per mg of protein. Protein was quantified with the Bradford method (Bradford, 1976) using the Bio-Rad reagent (Richmond, CA, USA) with BSA as a standard. Triton X-114 in a concentration of 1% does not influence the ELISA determinations and does not interfere with the protein quantification in tissue extracts (own results).

**Table 1** Antibodies employed in the different ELISAs, and the subgroup of antibodies which were used to compare with IHC

| PA comp. | Antibody[a] | Source[b] | Comparison | Conc. IHC[c] |
|---|---|---|---|---|
| uPA | Rb-PAb | DCE | + | 1.3 |
| | Ch-PAb | DCE | – | |
| tPA | Rb-PAb | DCE | + | 2.0 |
| | Ch-PAb | DCE | – | |
| PAI-1 | Rb-PAb | DCE | + | 1.0 |
| | Ch-PAb | DCE | – | |
| uPAR | Rb-PAb | FL | + | 1.5 |
| | Clone R2[d] | FL | – | |
| | Clone R3[d] | FL | – | |
| | Clone R5[d] | FL | – | |

[a] Rb = rabbit; Ch = chicken. PAb = polyclonal antibody. The R-clones are mouse monoclonal antibodies of the $IgG_1$ type. [b]DCE = Department of Chemical Endocrinology, University Hospital Nijmegen, The Netherlands (Grebenschikov et al, 1997); FL = Finsen Laboratory, Copenhagen, Denmark (Rønne et al, 1991, 1995). [c]Final concentration in µg $ml^{-1}$ as used for IHC. [d] R2 is directed against the non-ligand-binding part of the uPAR molecule. R3 and R5 are directed against the uPA-binding domain of uPAR (Rønne et al, 1991, 1995).

### IHC

Frozen sections of 4 µm thickness were fixed for 10 min in acetone at room temperature, and immunostaining was performed as described recently (Ferrier et al, 1998). Briefly, a three-step staining procedure with peroxidase-conjugated avidin–biotin complex (Vector Laboratories, Burlingame, CA, USA) was performed. The primary rabbit antibodies directed against uPA, tPA, PAI-1 and uPAR as listed in Table 1 were used; biotinylated goat anti-rabbit immunoglobulin (Vector Laboratories) was used as secondary antibody. Diaminobenzidine (Sigma, St Louis, MO, USA) was used as substrate to visualize the immune complexes.

To determine specificity of the immunostaining procedures, sections were incubated in the absence of the primary antibody. Specificity and sensitivity of the antibodies and procedures were also judged by staining negative and positive tissue control sections. Moreover, the specificity of most primary rabbit antibodies has previously been secured by affinity absorption studies, replacement with non-immune serum, and Western blotting analysis (Ferrier et al, 1998).

IHC results were analysed independently by two observers (CMF and GNPvM) without foreknowledge of the ELISA values. Discordances were resolved by joint review. The entire sections were reviewed and an overall semi-quantitative score, integrating the extent and intensity of staining of both tumour cells and stromal cells (fibroblasts, endothelial cells, inflammatory infiltrating cells as macrophages and lymphocytes), was assessed. Sections were graded from minimally 0 to maximally 3 as follows: 0, maximally 10% of the section stained weakly positive; 1, minimally 10% of the section stained weakly positive and maximally 10% of the section stained moderately or strongly positive; 2, 10–40% of the section stained moderately positive or 10–25% stained strongly positive; 3, over 40% of the section stained moderately positive or over 25% of the section stained strongly positive.

© Cancer Research Campaign 1999

### Statistical analysis

Spearman's rank correlations were evaluated for the relations between (i) ELISA values for each component as found in the two types of tissue extracts, (ii) ELISA values for the different components as found after extraction in detergent containing Tris buffer, (iii) IHC scores for the different components, and (iv) ELISA results and IHC scores for each of the four components tested.

The Kruskal–Wallis test for ranks was used for analysing differences of the mean ranks of ELISA values among the different scoring categories. *P*-values ≤ 0.05 were considered significant.

### RESULTS

### ELISA

Per lesion of the group of 33 tumour samples of various types, one set of ten consecutive 20-μm thick tissue sections was extracted with phosphate buffer and another set of such thick tissue sections was extracted with detergent-containing Tris buffer. The homogenates were analysed in the ELISAs for uPA, tPA, PAI-1 and uPAR. The detergent-containing Tris buffer extracted higher amounts of the analytes than the phosphate buffer. The two types of extracts yielded strongly correlating antigen level measurements for uPA (Spearman's rank correlation coefficient $r = 0.86$), PAI-1 ($r = 0.88$), tPA ($r = 0.86$) and uPAR ($r = 0.79$) with *P*-values ≤ 0.001.

For the compound group of cancer lesions, the results obtained per tumour type after extraction in the detergent-containing Tris buffer are presented in Table 2; these data were used for further comparisons with IHC. The levels found for uPA and PAI-1 were lowest in melanoma and breast carcinoma, and highest in lung carcinoma. The levels of tPA were generally low in all tumour types. uPAR levels were highest in colon and lung carcinoma and lowest in melanoma and breast cancer. Overall ELISA results in the compound group, the melanoma group and the breast cancer group are shown in Table 3.

**Table 4** Spearman's rank correlation coefficients (*r*) between PA components as determined by ELISA (right upper part of the Table) and by IHC (left lower part of the Table) in the compound group

| | uPA | tPA | PAI-1 | uPAR |
|---|---|---|---|---|
| uPA | | $r$ = –0.18<br>$P$ = 0.32 | $r$ = 0.65<br>$P$ = 0.0001 | $r$ = 0.74<br>$P$ = 0.0001 |
| tPA | $r$ = –0.22<br>$P$ = 0.22 | | $r$ = –0.38<br>$P$ = 0.03 | $r$ = –0.22<br>$P$ = 0.22 |
| PAI-1 | $r$ = 0.64<br>$P$ = 0.0001 | $r$ = –0.05<br>$P$ = 0.78 | | $r$ = 0.48<br>$P$ = 0.006 |
| uPAR | $r$ = 0.74<br>$P$ = 0.0001 | $r$ = –0.29<br>$P$ = 0.10 | $r$ = 0.58<br>$P$ = 0.0004 | |

For the compound group, the levels of uPA, PAI-1 and uPAR analysed in the extracts prepared with the detergent-containing Tris buffer intercorrelated significantly, whereas tPA levels correlated only with PAI-1 in a negative way. Correlations of tPA with the other components were not significant (Table 4). For the melanoma group, a significant correlation was found only between uPA and PAI-1. For the breast cancer group, significant correlations were found between uPA and PAI-1, uPA and uPAR and between uPA and tPA. Except that tPA correlated positively with PAI-1 in the melanoma group, all other intercorrelations had the same sign as shown in Table 4.

**Table 2** Descriptive statistics of ELISA results in the compound group. Results are shown per tumour type, obtained after extraction with detergent-containing Tris buffer. Medians and ranges (in brackets) are expressed in ng analyte mg$^{-1}$ protein

| | Lung ca, *n* = 6 | Cervical ca, *n* = 6 | Colon ca, *n* = 6 | Melanoma, *n* = 6 | Breast ca, *n = 9* |
|---|---|---|---|---|---|
| uPA | 6.28 (3.33–18.23) | 5.03 (2.33–8.72) | 3.65 (2.30–8.80) | 1.04 (0.62–1.77) | 2.67 (0.00–8.96) |
| tPA | 0.51 (0.18–1.07) | 1.01 (0.48–9.78) | 0.29 (0.13–1.02) | 0.43 (0.17–1.93) | 0.45 (0.21–8.05) |
| PAI-1 | 13.00 (3.75–21.85) | 6.46 (2.66–11.26) | 4.68 (1.41–10.28) | 2.65 (0.94–5.78) | 2.35 (1.27–7.71) |
| uPAR | 2.58 (1.39–5.63)[a] | 1.82 (1.04–3.42) | 2.58 (1.80–3.50) | 0.69 (0.44–3.68) | 1.02 (0.34–2.29) |

*n* = number of lesions analysed by ELISA; ca = carcinoma. [a]uPAR results are based on five lung carcinomas.

**Table 3** Descriptive statistics of the ELISA values obtained in the compound group, the melanoma and breast carcinoma group after extraction with detergent-containing Tris buffer. Data are expressed in ng analyte mg$^{-1}$ protein

| | 33 tumours, compound group | | | 23 melanoma lesions | | | 28 breast carcinoma lesions | | |
|---|---|---|---|---|---|---|---|---|---|
| | Mean ± s.d. | Median | Range | Mean ± s.d. | Median | Range | Mean ± s.d. | Median | Range |
| uPA | 4.19 ± 3.65 | 3.28 | 0.00–18.23 | 0.87 ± 0.55 | 0.80 | 0.16–1.88 | 2.00 ± 2.09 | 1.66 | 0.00–8.96 |
| tPA | 1.21 ± 2.21 | 0.48 | 0.13–9.78 | 0.45 ± 0.44 | 0.29 | 0.03–1.93 | 0.93 ± 1.68 | 0.36 | 0.05–8.05 |
| PAI-1 | 5.96 ± 5.01 | 4.55 | 0.94–21.85 | 2.68 ± 2.93 | 1.94 | 0.40–12.56 | 1.94 ± 1.99 | 1.35 | 0.14–7.71 |
| uPAR | 1.95 ± 1.26[a] | 1.84[a] | 0.34–5.63[a] | 2.09 ± 2.43 | 1.30 | 0.26–11.49 | 1.14 + 0.57 | 0.98 | 0.34–2.82 |

[a]Thirty-two instead of 33 tumour samples were analysed by ELISA.

 *© Cancer Research Campaign 1999*

**Table 5** IHC scoring results of the compound group, showing number of lesions per tumour type as classified in the particular scoring classes. Sections were stained with rabbit polyclonal antibodies

| | Score | Lung *n* = 6 | Cervix *n* = 6 | Colon *n* = 6 | Melanoma *n* = 6 | Breast *n* = 9 |
|---|---|---|---|---|---|---|
| uPA | 0 | 0 | 0 | 0 | 0 | 0 |
| | 1 | 0 | 0 | 0 | 3 | 3 |
| | 2 | 1 | 1 | 2 | 1 | 2 |
| | 3 | 5 | 5 | 4 | 2 | 4 |
| tPA | 0 | 2 | 1 | 2 | 1 | 4 |
| | 1 | 3 | 3 | 4 | 5 | 5 |
| | 2 | 1 | 2 | 0 | 0 | 0 |
| | 3 | 0 | 0 | 0 | 0 | 0 |
| PAI-1 | 0 | 0 | 0 | 0 | 0 | 0 |
| | 1 | 0 | 2 | 2 | 3 | 3 |
| | 2 | 2 | 2 | 4 | 1 | 3 |
| | 3 | 4 | 2 | 0 | 2 | 3 |
| uPAR | 0 | 0 | 0 | 0 | 0 | 0 |
| | 1 | 0 | 1 | 0 | 3 | 2 |
| | 2 | 4 | 2 | 4 | 2 | 4 |
| | 3 | 2 | 3 | 2 | 1 | 3 |

*n* = number of lesions tested for the specific tumour type.

**Table 6** IHC scoring results of melanoma and breast carcinoma groups. Distribution over the different scoring categories are given in percentages of the total of 23 melanoma and 28 breast carcinoma lesions

| | Melanoma/breast carcinoma | | | |
|---|---|---|---|---|
| | 0 | 1 | 2 | 3 |
| uPA | 0/0 | 35/36 | 52/36 | 13/28 |
| tPA | 17/36 | 53/61 | 17/3 | 13/0 |
| PAI-1 | 4/18 | 65/53 | 22/18 | 9/11 |
| uPAR | 0/0 | 22/7 | 30/39 | 48/54 |

**Table 7** Spearman's rank correlation coefficients (*r*) and *P*-values for the relationships between the ELISA values and the IHC scores as found in the compound group, the melanoma group and the breast carcinoma group

| | Compound group | | Melanoma | | Breast carcinoma | |
|---|---|---|---|---|---|---|
| | *r* | *P*-value | *r* | *P*-value | *r* | *P*-value |
| uPA | 0.68 | 0.0001 | 0.50 | 0.014 | 0.78 | 0.0001 |
| tPA | 0.54 | 0.0011 | 0.41 | 0.049 | 0.44 | 0.018 |
| PAI-1 | 0.66 | 0.0001 | 0.46 | 0.028 | 0.77 | 0.0001 |
| uPAR[a] | 0.60 | 0.0003 | 0.60 | 0.0026 | 0.49 | 0.0076 |

[a]The correlation coefficient of uPAR was based on 32 tumour samples.

### IHC

Sections adjacent to those analysed in the ELISAs were stained for uPA, tPA, PAI-1 and uPAR. The antibodies used were also incorporated in the ELISAs. In an earlier study we found that different primary antibodies (monoclonal and polyclonal) directed against the same component yielded comparable staining patterns, although intensities varied (Ferrier et al, 1998). The highest staining intensities were obtained with the rabbit polyclonal antibodies that were used for the present study. For the compound tumour group, scoring results are presented in Table 5. Table 6 summarizes the scoring results for the melanoma group and the breast carcinoma group. Figure 1 shows some examples of IHC stainings. Staining patterns differed among various tumour types and also among different cases of the same tumour type. Sections either showed isolated tumour cell positivity, isolated stromal cell positivity, complete negativity or combined positivity of stromal and tumour cells. Moreover, positivity was often heterogeneous within different areas of the same tumour sample. For uPA we found abundant stromal cell staining among all tested cancer types; tumour cell staining was seen very scarcely in breast carcinomas and colon carcinomas, whereas some of the melanomas, lung and cervical carcinomas showed abundant tumour cell reactivity. For PAI-1, substantial stromal cell staining was seen among all tumour types, whereas tumour cell staining was only prominent in some of the melanomas, breast and lung carcinomas. For tPA, the majority of cases showed weak immunoreactivity of tumour and/or stromal cells. uPAR stromal cell and tumour cell positivity was striking among all the tumour types. Control sections on which the first antibody was omitted showed no immunostaining, and all control tissue sections showed the correct staining pattern.

In the compound group, strong statistically significant correlations were found between the scores for uPA, PAI-1 and uPAR, whereas tPA did not correlate significantly with any of the other components (Table 4). For melanoma and breast carcinoma, significant correlations were found between uPA and PAI-1, and between uPA and uPAR. The correlation coefficients between tPA and the other components were negative for all tumour groups, whereas the other components intercorrelated positively.

### IHC versus ELISA

For the compound group of 33 tumour samples (32 samples for uPAR), the melanoma group and the breast carcinoma group, the IHC scores were compared with the ELISA values by calculation of Spearman correlation coefficients. Correlation coefficients varied between 0.41 and 0.78, and were all statistically significant (Table 7). Figures 2 and 3 show the IHC scores and the corresponding ELISA values, with the median ELISA value for each scoring category. For each group, the median ELISA value increased with increasing score category. All Kruskal–Wallis tests for comparison of the mean ranks of the ELISA values over the different IHC scoring categories were significant in the compound group. In the melanoma and breast carcinoma groups, these tests were significant for only some of the components (melanoma: for uPAR; breast carcinoma: for uPA, PAI-1 and uPAR).

### DISCUSSION

In the present study we compared the antigen levels of uPA, tPA, PAI-1 and uPAR in tissue extracts as determined by ELISA with the expression in corresponding IHC-stained cryostat sections as semi-quantitated by microscopic observation. Such an analysis was done on a compound group consisting of various tumour types, a melanoma group and a breast cancer group.

First we addressed the preparation of extracts for the ELISAs. We compared phosphate buffer and detergent-containing Tris

© Cancer Research Campaign 1999 

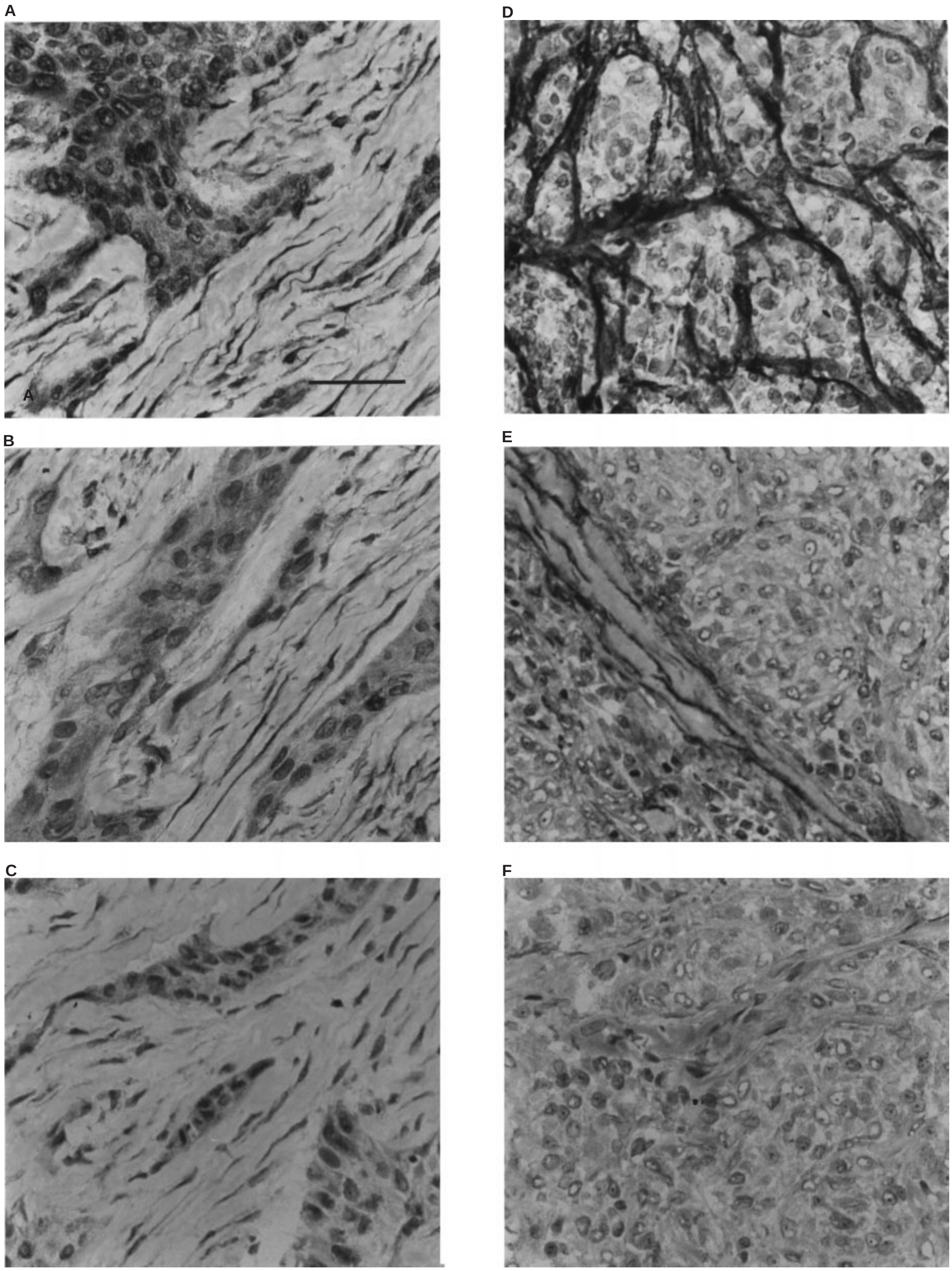

**Figure 1** IHC staining results showing different allocated scores and different distribution of positivity. Sections were stained with rabbit polyclonal antibodies. (**A, B, C**) A lung carcinoma stained for uPA, uPAR and tPA respectively. (**D, E, F**) A melanoma metastasis stained for PAI-1, uPAR and tPA respectively. (**A** and **D**) Sections with score = 3; (**B** and **E**) stainings with score = 2; (**C** and **E**) stainings with score = 1. (**A, B** and **C**) Show a combination of tumour cell and stromal cell positivity, with (**A**) strong and (**B** and **C**) moderate staining intensity. In (**C**) only a small fraction of tumour and stromal cells are positive. (**D**) Shows strong positivity of the extracellular matrix; (**E**) presents intense positivity of the stromal cells, but these positive cells cover the minority of the section. (**F**) Shows weak tumour cell staining. Bar = 50 µm, magnification is the same for all photographs. A colour reproduction of the Figure can be obtained from the corresponding author

*© Cancer Research Campaign 1999*

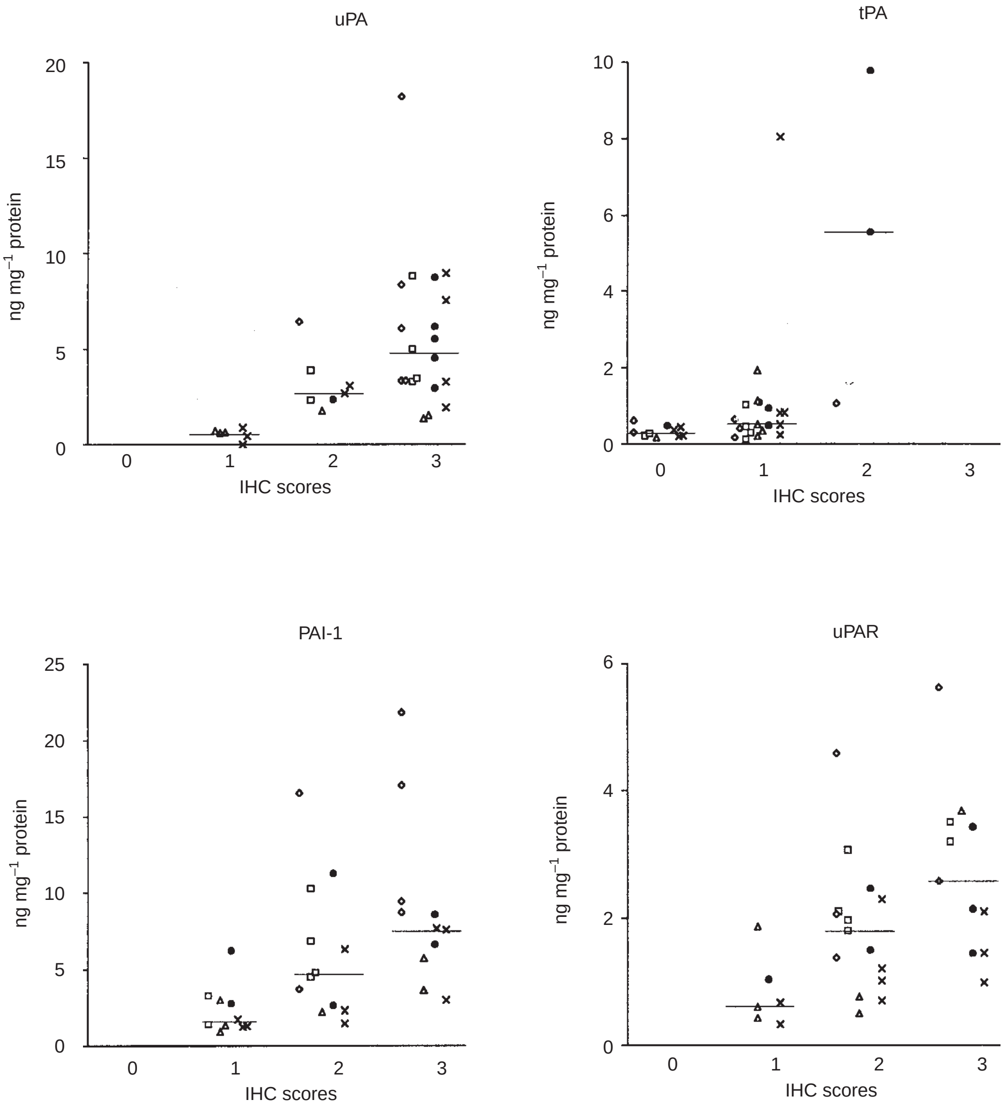

**Figure 2** Correlations between ELISA values and IHC scores for the compound group. ◇ = lung carcinoma; □ = colon carcinoma; △ = melanoma; ● = cervical carcinoma; × = breast carcinoma; —— = median value per scoring category buffer and found the latter most successful in extracting uPA, tPA, PAI-1 and uPAR out of tumour tissue. This is in agreement with previous observations (Rønne et al, 1995) and also with the concept that detergent-containing buffers are effective for extraction of membrane components and membrane-associated molecules (Behrendt et al, 1990; de Witte et al, 1997), as in our experiments for uPAR and uPA. Although the ELISAs were performed on extracts of a few tissue sections only (10 × 20 μm), all but one of the measurements were above the detection limit when the detergent-containing Tris buffer was used. This demonstrates the possibility to perform ELISAs on very small amounts of tissue, likely attributable to an efficient tissue extraction procedure and the use of sensitive ELISAs. For the comparison with IHC, we used the ELISA results obtained on extracts prepared with detergent-containing Tris buffer because of the high recovery obtained with this buffer for each of the components.

Both with ELISA and IHC, many correlations were found among the different PA components, which is in agreement with current knowledge (Rønne et al, 1995; Pappot et al, 1997).

Analysing the compound group of 33 lesions, we found correlations from 0.54 to 0.68 for uPA, tPA, PAI-1 and uPAR between the expression levels as determined by ELISA and IHC. Although these correlation coefficients are statistically highly significant, there is a partial overlap of ELISA values from the different scoring classes (Figure 2). Hence, for individual tumour cases the relation between ELISA and IHC is ambiguous. This indicates that the two techniques are not directly interchangeable and that their value for clinical purposes may be different. This was reconfirmed by the analysis of extended groups of melanoma and breast cancer lesions; these groups also showed considerable overlap (Figure 3). Correlation coefficients between ELISA and IHC results were of the same order as in the compound group in case of breast carcinoma and somewhat lower in case of melanoma.

We considered several aspects to optimize comparison between the results of the two techniques. Firstly, ELISA and IHC were performed on consecutive tissue sections which minimized the effect of tissue heterogeneity. To that end, IHC had to be done on frozen sections, although in common practice IHC will often be

© Cancer Research Campaign 1999

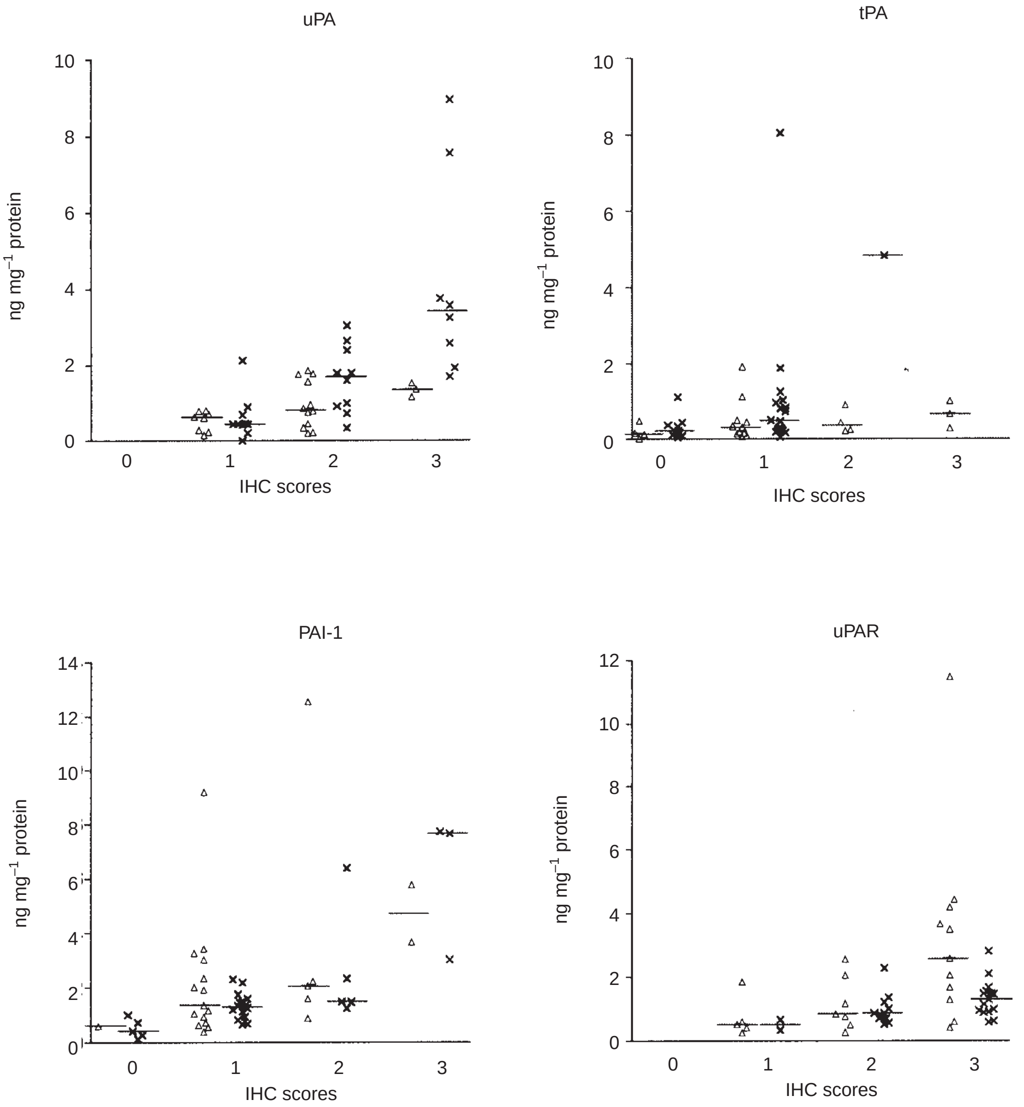

**Figure 3** Correlations between ELISA values and IHC scores for the melanoma and breast cancer group. △ = melanoma; × = breast carcinoma; —— = median value per scoring category performed on paraffin sections. Secondly, IHC was performed with antibodies that were also used in the ELISAs, which is advantageous to obtain good correlations because of uniform epitope recognition. Thirdly, both with ELISA and IHC all samples of the compound group were analysed in one run, evidently reducing inter-assay variations. The subsequently studied melanoma and breast carcinoma samples were also analysed in one run.

Complete agreement was not attained despite the considerations mentioned above. A possible explanation for the discrepancies is that fractions of a component, as a result of divergent presentation of epitopes in tumour tissue extracts and in tissue sections, may be detected with different efficiencies by ELISA and IHC. For example, during tissue processing new complexes between components may form while pre-existing complexes may dissociate (de Witte et al, 1997; Pappot et al, 1997). Moreover, both the catching and detecting antibodies in the ELISAs exert selection, whereas in IHC only one antibody selects the antigenic determinants. Another point is that, in IHC, estimation of protein expression levels above the level causing maximum staining is impossible, whereas with ELISA discrimination is possible even among very high analyte concentrations.

Only a few other studies have paid attention to the relationship between ELISA and IHC for one or more components of the PA system. Significant correlations between IHC scores and ELISA values were found for uPA in primary breast carcinoma lesions (Jänicke et al, 1990) and colon carcinoma (Sier et al, 1991), for uPA and PAI-1 in cervical cancer of the uterus (Kobayashi et al, 1994) and lymph node metastases of breast cancer but not in primary breast cancer (Christensen et al, 1996), and for PAI-1 but not for uPAR in non-small-cell lung cancer (Pappot et al, 1997). Some of the above mentioned studies described IHC on paraffin sections (Jänicke et al, 1990; Sier et al, 1991; Kobayashi et al, 1994) whereas others, like us, performed IHC on frozen tissue (Christensen et al, 1996; Pappot et al, 1997). Correlation coefficients reported range from 0.49 to 0.80. However, the correlation coefficients found by different groups are difficult to compare, among others since different IHC scoring methods and different statistical evaluations were used.

 *© Cancer Research Campaign 1999*

At present, ELISA methods are preferred for assessment of the PA components, at least by their more extensively proven clinical value, unequivocal interpretation and the ongoing of quality assurance programmes. Nevertheless, the insight into tissue distribution and the ample availability of paraffin tissue render that ultimately both IHC and ELISA may be used complementarily, depending on the amount of tissue and the way of fixation available. Future studies comparing the prognostic impact of both approaches should further position the two techniques in the context of clinical use.

### ACKNOWLEDGEMENTS

The Dutch Cancer Society financially supported this study (project number NKB-KWF 94-722). We gratefully acknowledge Dr N Brünner (Finsen Laboratory, Copenhagen, Denmark) for providing antibodies against uPAR which were used for IHC and in the ELISA for uPAR. This work has been performed in collaboration with the Concerted European Action BIOMED-1 research group on 'Clinical Relevance of Proteases in Tumour Invasion and Metastasis'.

### REFERENCES

Andreasen PA, Kjøller L, Christensen L and Duffy MJ (1997) The urokinase-type plasminogen activator system in cancer metastasis: a review. *Int J Cancer* **72**: 1–22

Behrendt N, Rønne E, Ploug M, Petri T, Løber D, Nielsen LS, Schleuning W-D, Blasi F, Appella E and Danø K (1990) The human receptor for urokinase plasminogen activator. $NH_2$-terminal amino acid sequence and glycosylation variants. *J Biol Chem* **265**: 6453–6460

Bradford MM (1976) A rapid and sensitive method for the quantitation of microgram quantities of protein utilizing the principle of protein-dye binding. *Anal Biochem* **72**: 248–254

Christensen L, Wiborg Simonsen AC, Heegaard CW, Moestrup SK, Andersen JA and Andreasen PA (1996) Immunohistochemical localization of urokinase-type plasminogen activator, type-1 plasminogen-activator inhibitor, urokinase receptor and $\alpha_2$-macroglobulin receptor in human breast carcinomas. *Int J Cancer* **66**: 441–452

de Vries TJ, van Muijen GNP and Ruiter DJ (1996) The plasminogen activation system in tumour invasion and metastasis. *Path Res Pract* **192**: 718–733

de Witte H, Pappot H, Brünner N, Grøndahl-Hansen J, Høyer-Hansen G, Behrendt N, Guldhammer-Skov B, Sweep F, Benraad T and Danø K (1997) ELISA for complexes between urokinase-type plasminogen activator and its receptor in lung cancer tissue extracts. *Int J Cancer* **72**: 416–423

Duggan C, Maguire T, McDermott E, O'Higgins N, Fennelly JJ and Duffy MJ (1995) Urokinase plasminogen activator and urokinase plasminogen activator receptor in breast cancer. *Int J Cancer* **61**: 597–600

Ferrier CM, van Geloof WL, de Witte JH, Kramer MD, Ruiter DJ and van Muijen GNP (1998) Epitopes of components of the plasminogen activation system are re-exposed in formalin-fixed paraffin sections by different retrieval techniques. *J Histochem Cytochem* **46**: 469–476

Foekens JA, Buessecker F, Peters HA, Krainick U, van Putten WLJ, Look MP, Klijn JGM and Kramer MD (1995) Plasminogen activator inhibitor-2: prognostic relevance in 1012 patients with primary breast cancer. *Cancer Res* **55**: 1423–1427

Ganesh S, Sier CFM, Heerding MM, van Krieken JHJM, Griffioen G, Welvaart K, van de Velde CJH, Verheijen JH, Lamers CBHW and Verspaget HW (1996) Prognostic value of the plasminogen activation system in patients with gastric carcinoma. *Cancer* **77**: 1035–1043

Grebenschikov N, Geurts-Moespot A, de Witte H, Heuvel J, Leake R, Sweep F and Benraad T (1997) A sensitive and robust assay for urokinase and tissue-type plasminogen activators (uPA and tPA) and their inhibitor type I (PAI-1) in breast tumor cytosols. *Int J Biol Markers* **12**: 6–14

Heiss MM, Babic R, Allgayer H, Gruetzner KU, Jauch K-W, Loehrs U and Schildberg FW (1996) The prognostic impact of the urokinase-type plasminogen activator system is associated with tumour differentiation in gastric cancer. *Eur J Surg Oncol* **22**: 74–77

Hsu DW, Efird JT and Hedley-Whyte ET (1995) Prognostic role of urokinase-type plasminogen activator in human gliomas. *Am J Pathol* **147**: 114–123

Jänicke F, Schmitt M, Hafter R, Hollrieder A, Babic R, Ulm K, Gössner W and Graeff H (1990) Urokinase-type plasminogen activator (u-PA) antigen is a predictor of early relapse in breast cancer. *Fibrinolysis* **4**: 69–78

Kobayashi H, Fujishiro S and Terao T (1994) Impact of urokinase-type plasminogen activator and its inhibitor type 1 on prognosis in cervical cancer of the uterus. *Cancer Res* **54**: 6539–6548

Kuhn W, Pache L, Schmalfeldt B, Dettmar P, Schmitt M, Jänicke F and Graeff H (1994) Urokinase (uPA) and PAI-1 predict survival in advanced ovarian cancer patients (FIGO III) after radical surgery and platinum-based chemotherapy. *Gynecol Oncol* **55**: 401–409

Mulcahy HE, Duffy MJ, Gibbons D, McCarthy P, Parfrey NA, O'Donoghue DP and Sheahan K (1994) Urokinase-type plasminogen activator and outcome in Dukes' B colorectal cancer. *Lancet* **344**: 583–584

Pappot H, Guldhammer Skov B, Pyke C and Grøndahl-Hansen J (1997) Levels of plasminogen activator inhibitor type 1 and urokinase plasminogen activator receptor in non-small cell lung cancer as measured by quantitative ELISA and semiquantitative immunohistochemistry. *Lung Cancer* **17**: 197–209

Rønne E, Behrendt N, Ellis V, Ploug M, Danø K and Høyer-Hansen G (1991) Cell-induced potentiation of the plasminogen activation system is abolished by a monoclonal antibody that recognizes the $NH_2$-terminal domain of the urokinase receptor. *FEBS Lett* **288**: 233–236

Rønne E, Høyer-Hansen G, Brünner N, Pederson H, Rank F, Osborne CK, Clark GM, Danø K and Grøndahl-Hansen J (1995) Urokinase receptor in breast cancer tissue extracts. Enzyme-linked immunosorbent assay with a combination of mono- and polyclonal antibodies. *Breast Cancer Res Treat* **33**: 199–207

Ruiter DJ, Ferrier CM, van Muijen GNP, Henzen-Logmans SC, Kennedy S, Kramer MD, Nielsen BS and Schmitt M (1998) Quality control of immunohistochemical evaluation of tumour-associated plasminogen activators and related components. *Eur J Cancer* **34**: 1334–1340

Schmitt M, Thomssen C, Ulm K, Seiderer A, Harbeck N, Höfler H, Jänicke F and Graeff H (1997) Time-varying prognostic impact of tumour biological factors urokinase (uPA), PAI-1 and steroid hormone receptor status in primary breast cancer. *Br J Cancer* **76**: 306–311

Sier CFM, Fellbaum C, Verspaget HW, Schmitt M, Griffioen G, Graeff H, Höfler H and Lamers CBHW (1991) Immunolocalization of urokinase-type plasminogen activator in adenomas and carcinomas of the colorectum. *Histopathology* **19**: 231–237

Sweep CGJ, Geurts-Moespot J, Grebenschikov N, de Witte JH, Heuvel JJTM, Schmitt M, Duffy MJ, Jänicke F, Kramer MD, Foekens JA, Brünner N, Brugal G, Pedersen AN and Benraad TJ (1998) External quality assessment of trans-European multicentre antigen determinations (Enzyme-linked immunosorbent assay) of urokinase-type plasminogen activator (uPA) and its type 1 inhibitor (PAI-1) in human breast cancer tissue extracts. *Br J Cancer* **78**: 1434–1441

Thorpe SM (1987) Steroid receptors in breast cancer: sources of inter-laboratory variation in dextran-charcoal assays. *Breast Cancer Res Treat* **9**: 175–189

Umeda T, Eguchi Y, Okino K, Kodama M and Hattori T (1997) Cellular localization of urokinase-type plasminogen activator, its inhibitors, and their mRNAs in breast cancer tissues. *J Pathol* **183**: 388–397

© Cancer Research Campaign 1999
